# Resveratrol Alleviates Acute *Campylobacter jejuni* Induced Enterocolitis in a Preclinical Murine Intervention Study

**DOI:** 10.3390/microorganisms8121858

**Published:** 2020-11-25

**Authors:** Markus M. Heimesaat, Soraya Mousavi, Ulrike Escher, Fábia Daniela Lobo de Sá, Elisa Peh, Jörg-Dieter Schulzke, Sophie Kittler, Roland Bücker, Stefan Bereswill

**Affiliations:** 1 Institute of Microbiology, Infectious Diseases and Immunology, Charité—University Medicine Berlin, Corporate Member of Freie Universität Berlin, Humboldt-Universität zu Berlin, and Berlin Institute of Health, 12203 Berlin, Germany; soraya.mousavi@charite.de (S.M.); ulrike.escher@charite.de (U.E.); stefan.bereswill@charite.de (S.B.); 2 Institute of Clinical Physiology, Department of Gastroenterology, Infectious Diseases and Rheumatology, Charité—Universitätsmedizin Berlin, Corporate Member of Freie Universität Berlin, Humboldt-Universität zu Berlin, and Berlin Institute of Health, 12203 Berlin, Germany; fabia.lobo-da-fonseca@charite.de (F.D.L.d.S.); joerg.schulzke@charite.de (J.-D.S.); roland-felix.buecker@charite.de (R.B.); 3 Institute for Food Quality and Food Safety, University of Veterinary Medicine Hannover, Foundation, 30559 Hannover, Germany; Elisa.Peh@tiho-hannover.de (E.P.); sophie.kittler@tiho-hannover.de (S.K.)

**Keywords:** polyphenols, resveratrol, preclinical intervention study, *Campylobacter jejuni*, secondary abiotic IL-10^−/−^ mice, acute campylobacteriosis model, pro-inflammatory immune responses, host-pathogen interactions, intestinal epithelial barrier function

## Abstract

The polyphenolic compound resveratrol has been shown to exert health-beneficial properties. Given globally emerging *Campylobacter* infections in humans, we addressed potential anti-pathogenic, immuno-modulatory and intestinal epithelial barrier preserving properties of synthetic resveratrol in the present preclinical intervention study applying a murine acute campylobacteriosis model. Two days following peroral *C. jejuni* infection, secondary abiotic IL-10^−/−^ mice were either subjected to resveratrol or placebo via the drinking water. Whereas placebo mice suffered from acute enterocolitis at day 6 post-infection, resveratrol treatment did not only lead to improved clinical conditions, but also to less pronounced colonic epithelial apoptosis as compared to placebo application. Furthermore, *C. jejuni* induced innate and adaptive immune cell responses were dampened in the large intestines upon resveratrol challenge and accompanied by less colonic nitric oxide secretion in the resveratrol versus the placebo cohort. Functional analyses revealed that resveratrol treatment could effectively rescue colonic epithelial barrier function in *C. jejuni* infected mice. Strikingly, the disease-alleviating effects of resveratrol could additionally be found in extra-intestinal and also systemic compartments at day 6 post-infection. For the first time, our current preclinical intervention study provides evidence that peroral resveratrol treatment exerts potent disease-alleviating effects during acute experimental campylobacteriosis.

## 1. Introduction

*Campylobacter* infections are emerging worldwide and cause tremendous health burdens and socioeconomic costs, irrespective whether low-, middle- or high-income countries are concerned [1,2]. Among the *Campylobacter* genus, *C. jejuni* are the most prevalent species causing human illnesses [2]. The Gram-negative bacteria reside as commensals in the gut microbiota of, clinically, mostly unaffected warm-blooded vertebrate species including livestock such as poultry. The enteropathogens might be transferred to humans, however, following ingestion of contaminated undercooked meat or surface water [3,4]. Following successful passage of the stomach, adhesion to and invasion of the intestinal epithelia by *C. jejuni*, innate and adaptive immune cells are recruited to the colonic mucosa and lamina propria. In turn, pro-inflammatory mediator secretion leads to oxidative stress to and apoptosis of colonic epithelial cells mounting in epithelial barrier dysfunction [5,6].

Following an incubation period of between 2 and 5 days, *C. jejuni* induced symptoms might arise, and severity of disease ranges from relatively mild discomfort to acute campylobacteriosis characterized by fever, abdominal cramps, watery or even inflammatory and bloody diarrhea with mucous discharge depending on the enteropathogen’s virulence factors and the host immune status [2,7]. Infected intestinal tissue samples display inflammatory changes including immune cell infiltrates within the mucosa and lamina propria, crypt abscesses, epithelial cell apoptosis and ulcerations [6]. In most instances, the course of disease is benign and self-limiting, and patients require, if at all, symptomatic treatment such as substitution of electrolytes. Antibiotic treatment may be required, however, in immunocompromised patients suffering from severe campylobacteriosis [2,8]. In rare instances, even post-infectious autoimmune diseases such as reactive arthritis, Guillain Barré and Miller Fisher syndromes and intestinal morbidities including chronic inflammatory bowel diseases, irritable bowel syndrome and celiac disease may develop with a latency of weeks to months post-infection (p.i.) [2,8].

Despite the emergence of human campylobacteriosis, the molecular mechanisms underlying pathogen–host interactions are only incompletely understood. In order to tackle this limitation, our group has established a murine infection and inflammation model resembling key features of severe campylobacteriosis in humans. Therefore, the commensal microbiota was depleted in IL-10^−/−^ mice by broad-spectrum antibiotic treatment in order to override the physiological colonization resistance caused by the murine gut microbiota and, hence, to facilitate stable *C. jejuni* infection [9]. Furthermore, since mice are approximately 10,000 times more resistant to bacterial lipooligosaccharide (LOS) and lipopolysacchride (LPS) as compared to humans [10], and *il10* gene deficiency renders the murine host susceptible to pathogenic LOS and LPS, peroral *C. jejuni* infection of secondary abiotic IL-10^−/−^ mice resulted in nonself-limiting acute enterocolitis with extra-intestinal and even systemic inflammatory manifestations within one week due to LOS-induced and Toll-like receptor-4 (TLR-4)-dependent immune responses [9,11]. Our murine *C. jejuni* infection and inflammation model has been proven reliable for testing molecules and compounds of interest regarding their anti-pathogenic and immuno-modulatory properties as shown in our recent preclinical intervention studies [12,13,14,15,16,17].

Resveratrol (3,5,4‘-trihydroxy-trans-stilbene) constitutes a polyphenolic molecule that can be found in the skin and seeds of grapes, in red wine, in various fruits and in peanuts, for instance, acting as phytoalexin against pathogens such as bacteria and fungi [18,19]. Several in vitro and in vivo studies revealed pleiotropic health-beneficial properties of resveratrol such as anti-oxidant, anti-inflammatory, anti-microbial, anti-cancer, anti-diabetic, anti-dyslipidemic, anti-aging, cardio-protective and neuro-protective effects as reviewed in detail previously [18,19]. In our recent study we showed that resveratrol treatment could ameliorate acute small intestinal inflammation by down-regulating T helper-1 (Th-1) cell immune responses following peroral *Toxoplasma gondii* infection. This prompted us to test resveratrol in our present preclinical intervention study for its anti-pathogenic and immuno-modulatory effects applying another acute and fatal intestinal inflammation model of different (i.e., enteropathologic) etiology and manifestation in a different intestinal (i.e., large intestinal) compartment.

## 2. Materials and Methods

### 2.1. Ethics Approval

Mouse experiments were approved by the local ethical authorities (“Landesamt für Gesundheit und Soziales”, LaGeSo, Berlin, Germany; registration numbers G0109/19, approved on 15 July 2019) and performed in accordance to the European Guidelines for animal welfare (2010/63/EU). The clinical conditions of mice were surveyed twice daily.

### 2.2. Determination of the Minimal Inhibitory Concentration of Resveratrol

For the assessment of the minimal inhibitory concentration (MIC) values of resveratrol, 20 *C. jejuni* isolates including the reference strain 81-176 used for murine infection in the present study (see below) were tested for their antimicrobial susceptibility applying the broth microdilution method. Procedures regarding inoculum density, growth medium, incubation time and conditions were performed according to the recommendations indicated in the Clinical and Laboratory Standards Institute (CLSI) document VET01-A5 [20]. Twofold serial dilutions of resveratrol ranging from 0.008 to 8.0 mmol/L (1.78–1826 mg/mL) were tested. Stock solutions were prepared in Mueller–Hinton broth and adjusted to pH 7.3.

### 2.3. Generation of Secondary Abiotic IL-10^−/−^ Mice

IL-10^−/−^ mice (C57BL/6j background) were reared under specific pathogen-free conditions in the identical housing room located at the Forschungseinrichtungen für Experimentelle Medizin (Charité—University Medicine Berlin). Within an experimental semi-barrier mice were maintained in cages with filter tops under standard conditions (12 h light/12 dark cycle, 22–24 °C room temperature, 55 ± 15% humidity) and had unlimited access to autoclaved standard food pellets (ssniff R/M-H, V1534-300, Sniff, Soest, Germany). In order to assure stable gastrointestinal *C. jejuni* colonization by counteracting physiological colonization resistance [21], microbiota-depleted (i.e., secondary abiotic) mice were generated as stated earlier [21,22]. In brief, 3-week-old mice were challenged with an 8-week course of broad-spectrum antibiotic treatment. Therefore, five different antibiotic compounds including imipenem (250 mg/L; Fresenius Kabi, Bad Homburg, Germany), vancomycin (500 mg/L; Hikma Pharmaceuticals, London, UK), metronidazole (1 g/L; B. Braun, Melsungen, Germany), ampicillin plus sulbactam (1 g/L; Dr. Friedrich Eberth Arzneimittel, Ursensollen, Germany), and ciprofloxacin (200 mg/L; Fresenius Kabi, Bad Homburg, Germany) were added to autoclaved tap water (ad libitum) as reported by us earlier [22]. The antibiotic cocktail was withdrawn three days before infection in order to assure antibiotic washout. All mice were maintained and handled under strict aseptic precautions throughout the entire experiment.

### 2.4. Resveratrol Treatment

Treatment with synthetic resveratrol (Sigma-Aldrich, München, Germany) started two days after the initial *C. jejuni* infection. Resveratrol was dissolved in 2% carboxy-methyl-cellulose (to a final concentration of 0.05%) and provided to mice by adding the solution to the autoclaved tap water. The final concentration of the resveratrol solution was 300 mg/L (resulting dose of 60 mg per kg body weight per day). Placebo control animals received vehicle only.

### 2.5. C. jejuni Infection and Gastrointestinal Colonization

Four-month-old sex- and age-matched littermate mice were orally challenged with 10^9^ viable *C. jejuni* strain 81-176 cells on day (d) 0 and d1 by gavage. For surveying pathogenic colonization efficacies, *C. jejuni* were cultured from serial dilutions of fecal samples taken during the course of infection and, additionally, of the colon, terminal ileum, duodenum and stomach lumen upon necropsy at d6 p.i. as reported earlier [21]. The detection limit of the *C. jejuni* load was 100 colony-forming units per g (CFU/g).

### 2.6. Clinical Conditions

The clinical conditions of mice were monitored before and after *C. jejuni* infection on the basis of a standardized clinical scoring system (maximum 12 points) addressing the clinical aspect/wasting (0: normal; 1: ruffled fur; 2: less locomotion; 3: isolation; 4: severely compromised locomotion, pre-final aspect), fecal consistency (0: formed feces; 2: pasty feces; 4: liquid feces) and abundance of blood in the feces (0: no blood; 2: microscopic detection of blood by the Guajac method using Haemoccult, Beckman Coulter/PCD, Germany; 4: macroscopic blood visible) as described earlier [23].

### 2.7. Sampling Procedures

At d6 p.i., mice were sacrificed by CO_2_ asphyxiation. Luminal samples from the colon, terminal ileum, duodenum and stomach and ex vivo biopsies from the liver, kidneys and colon as well as cardiac blood were derived under aseptic precautions. Colonic and extra-intestinal samples were taken from each mouse in parallel for subsequent immunological, immunohistopathological and microbiological analyses.

### 2.8. Immunohistochemistry

Quantitative in situ immunohistochemical analyses were done with 5% formalin fixed and paraffin-embedded colonic tissue samples [24,25]. In brief, in order to detect apoptotic epithelial cells, macrophages/monocytes, T and B lymphocytes, 5 μm thin colonic paraffin sections were stained with primary antibodies against cleaved caspase 3 (Asp175, Cell Signaling, Beverly, MA, USA, 1:200), F4/80 (# 14-4801, clone BM8, eBioscience, San Diego, CA, USA, 1:50), CD3 (#N1580, Dako, Glostrup, Denmark, 1:10), and B220 (#14-0452-81, eBioscience; 1:200), respectively. Positively stained cells were examined by light microscopy (magnification 100× and 400×), and for each mouse the average number of respective positively stained cells was determined within six high-power fields (HPF, 0.287 mm^2^, 400× magnification) by a blinded investigator.

### 2.9. Measurements of Intestinal, Extra-Intestinal and Systemic Inflammatory Mediators

After longitudinal cut, large intestinal explants were washed in phosphate-buffered saline (PBS; Gibco, Life Technologies, Loughborough, UK), and strips of colonic and liver tissues (both approximately 1 cm^2^) and one kidney (cut longitudinally) were placed in flat-bottom 24-well culture plates (Nunc, Darmstadt, Germany) containing 500 μL serum-free RPMI 1640 medium (Thermo Fisher Scientific, Waltham, MA, USA) supplemented with penicillin (100 U/mL) and streptomycin (100 µg/mL, Biochrom, Berlin, Germany). After 18 h at 37 °C, respective culture supernatants as well as serum samples were tested for tumor necrosis factor-α (TNF-α), interferon (IFN)-γ and interleukin (IL)-6 by the Mouse Inflammation Cytometric Bead Assay (CBA; BD Biosciences, Heidelberg, Germany) on a BD FACSCanto II flow cytometer (BD Biosciences, Heidelberg, Germany). Nitric oxide concentrations were determined by the Griess reaction as described earlier [22].

### 2.10. Electrophysiological Measurements

Ex vivo biopsies were taken from the terminal large intestines and transferred to Ussing chambers (remaining unstripped; 0.049 cm^2^ area). The transmural electrical resistance (R^t^) was measured with an automatic clamp device (CVC6, Fiebig Hard & Software, Berlin, Germany) under voltage clamp conditions for one hour at 37 °C. The composition of the with carbogen gas (pH 7.4) equilibrated bathing solution was as follows: L-glutamine (2.5 mmol/L), beta-hydroxybutyric acid (0.5 mmol/L), D(+)-glucose (10.0 mmol/L), D(+)-mannose (10.0 mmol/L), NaCl (113.6 mmol/L), KCl (5.4 mmol/L), CaCl_2_ (1.2 mmol/L), MgCl_2_ (1.2 mmol/L), Na_2_HPO_4_ (2.4 mmol/L), NaH_2_PO_4_ (0.6 mmol/L) and NaHCO_3_ (21.0 mmol/L).

### 2.11. Statistical Analysis

The significance levels (with *p* values ≤ 0.05 indicative for significant differences) were calculated with the following tests (applying GraphPad Prism v7, San Diego, CA, USA): Mann–Whitney test (pairwise comparison, non-normal data distribution); one-sided ANOVA test and Tukey post-correction (multiple comparisons; normal data distribution); the Kruskal–Wallis test and Dunn’s post-correction (multiple comparisons; non-normal data distribution). Data sets were generated from four independent experiments.

## 3. Results

### 3.1. Antimicrobial Properties of Resveratrol against C. jejuni In Vitro

First, we investigated potential antimicrobial effects of resveratrol directed against *C. jejuni*. In vitro studies with 20 *C. jejuni* isolates including the reference strain 81-176 revealed a resveratrol MIC_90_ value of 228.25 mg/L (1.0 mmol/L) with MICs ranging from 57.1 mg/L (0.25 mmol/L) to 456.5 mg/L (2.0 mmol/L) (Supplementary Figure S1), whereas the *C. jejuni* strain 81-176 yielded an MIC value of 456.5 mg/L (2.0 mmol/L).

### 3.2. Gastrointestinal Pathogen Loads Following Resveratrol Treatment of Mice Suffering from C. jejuni Induced Enterocolitis

**Figure 1:**
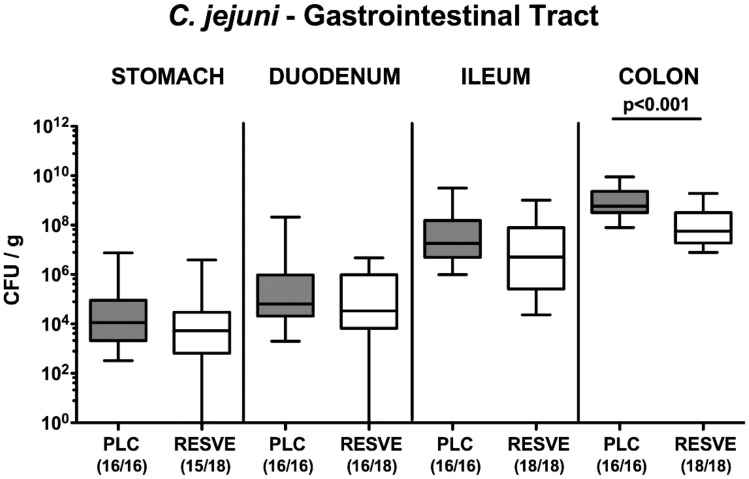
Gastrointestinal *C. jejuni* loads following resveratrol treatment of infected mice suffering from acute enterocolitis. Starting two days after initial peroral infection with *C. jejuni* strain 81-176 on day (d) 0 and d1, secondary abiotic IL-10^−/−^ mice were treated with resveratrol (RESVE; white boxes) or placebo (PLC; grey boxes) via the drinking water. On d6 post-infection, *C. jejuni* loads were quantitatively assessed in distinct parts of the gastrointestinal tract by culture (in colony-forming units (CFU) per g feces). Box plots represent the 75th and 25th percentiles of the median (black bar inside the boxes). Total range, significance levels (*p* values as determined by the Mann–Whitney test) and the number of culture-positive mice out of the total number of analyzed animals (in parentheses) are given. Data were pooled from three independent experiments.

Following peroral infection with 10^9^ viable *C. jejuni* strain 81-176 cells on d0 and d1, the pathogen could stably establish within the intestinal tract of secondary abiotic IL-10^−/−^ mice from either cohort as indicated by median fecal loads of approximately 10^9^ CFU per g (Supplementary Figure S2). On d3, d5 and d6 p.i., however, fecal *C. jejuni* loads were slightly lower (up to one log order of magnitude) in resveratrol- as compared to placebo-treated mice (*p* < 0.05–0.001; Supplementary Figure S2). Furthermore, upon necropsy we surveyed the pathogen densities alongside the gastrointestinal tract. Whereas the stomach, duodenum and ileum of resveratrol- and placebo-treated mice harbored comparable pathogen numbers, one order of magnitude lower *C. jejuni* counts could be assessed in luminal samples taken from the colon in the former as compared to the latter on d6 p.i. (*p* < 0.001; Figure 1). Hence, resveratrol-treated mice harbored slightly lower *C. jejuni* cell counts in their large intestines as compared to placebo controls.

### 3.3. Clinical Conditions of Resveratrol-Treated Mice Suffering from C. jejuni Induced Enterocolitis

**Figure 2:**
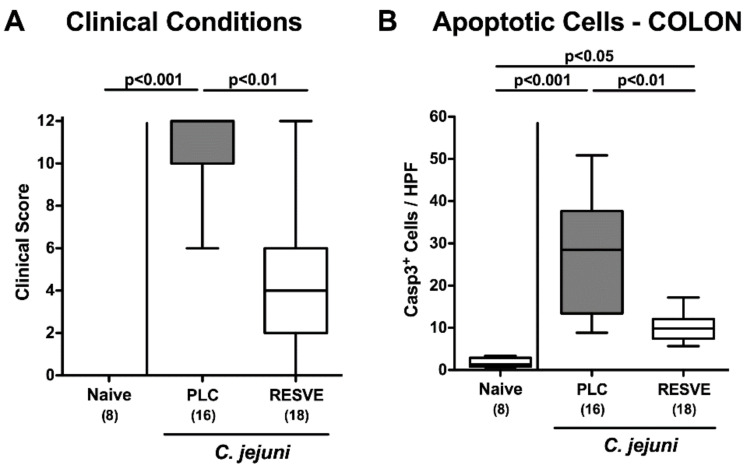
Clinical conditions and microscopic inflammatory responses following resveratrol treatment of infected mice suffering from acute enterocolitis. Starting two days after initial peroral infection with *C. jejuni* strain 81-176 on d0 and d1, secondary abiotic IL-10^−/−^ mice were treated with resveratrol (RESVE; white boxes) or placebo (PLC; grey boxes) via the drinking water. On d6 post-infection, (**A**) clinical conditions were quantitatively assessed applying a clinical scoring system. Furthermore, (**B**) apoptotic cells (cleaved caspase3^+^, Casp3^+^) were assessed microscopically from six high-power fields (HPF, 400× magnification) per animal in immunohistochemically stained colonic paraffin sections. Naive mice served as untreated and uninfected controls. Box plots represent the 75th and 25th percentiles of the median (black bar inside the boxes). Total range, significance levels (*p* values as determined by the Kruskal–Wallis test followed by Dunn’s correction) and numbers of mice (in parentheses) are given. Data were pooled from three independent experiments.

We further assessed the clinical outcome of *C. jejuni* infection over time by applying a standardized scoring system by which wasting, severity of diarrhea and abundance of fecal blood could be quantified. Within six days, *C. jejuni* infected mice from the placebo cohort developed acute enterocolitis as indicated by wasting and bloody diarrhea, whereas in resveratrol-treated mice clinical signs were less pronounced as early as d5 p.i. (*p* < 0.01; Supplementary Figure S3). At the end of the observation period, median clinical scores of 12 and 4 could be determined in the placebo and resveratrol cohorts, respectively (*p* < 0.01; Figure 2A). Notably, the standard deviation in the resveratrol group was relatively high, given that some mice were suffering from full blown disease, whereas others displayed only mild or even no clinical signs of *C. jejuni* infection at all (Figure 2A). Hence, resveratrol treatment resulted in a better clinical outcome of *C. jejuni* induced disease.

### 3.4. Apoptotic Responses in Large Intestinal Epithelia of Resveratrol-Treated Mice Suffering from C. jejuni Induced Enterocolitis

Given that apoptosis is regarded a reliable parameter for the grading of intestinal inflammation [21], we quantitatively assessed apoptotic cells in colonic epithelia applying in situ immunohistochemistry. In fact, *C. jejuni* infection was associated with multifold increases in cleaved caspase3^+^ cell numbers in colonic epithelia (*p* < 0.05–0.001 versus naive; Figure 2B, Supplementary Figure S4A). These increases, however, were far less pronounced following resveratrol as compared to placebo treatment at d6 p.i. (*p* < 0.01; Figure 2B, Supplementary Figure S4A). Hence, ameliorated gross *C. jejuni* induced disease was accompanied by dampened apoptotic cell responses in the large intestinal epithelia upon resveratrol treatment.

### 3.5. Inflammatory Immune Responses in the Large Intestines of Resveratrol-Treated Mice Suffering from C. jejuni Induced Enterocolitis

**Figure 3:**
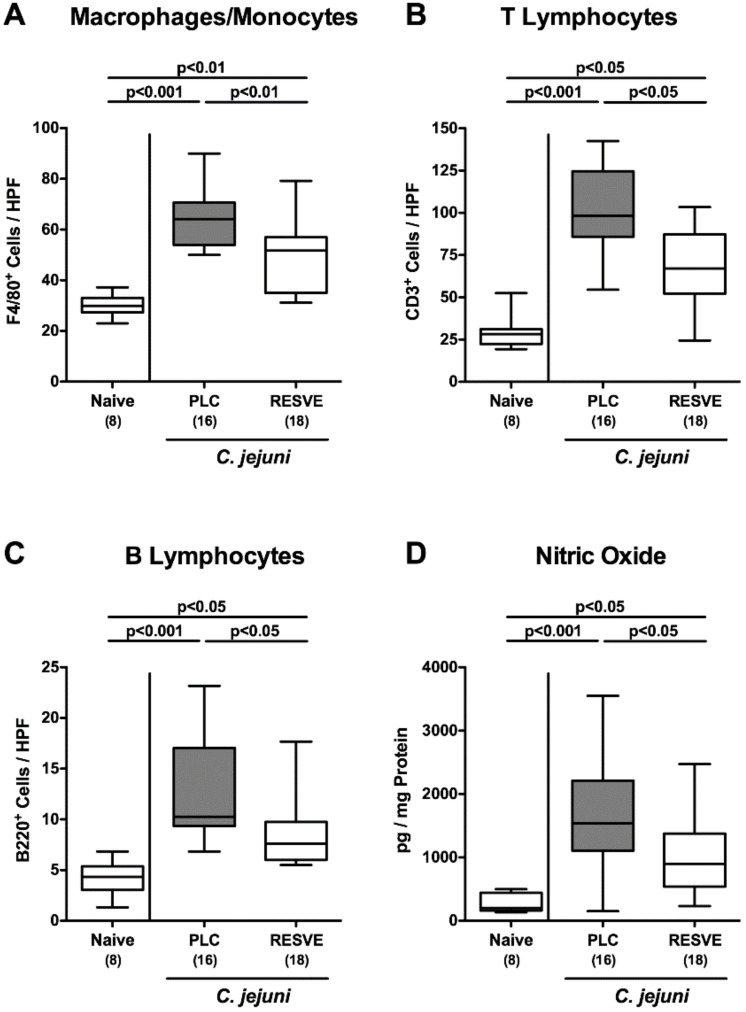
Inflammatory immune responses upon resveratrol treatment of infected mice suffering from acute enterocolitis. Starting two days after initial peroral infection with *C. jejuni* strain 81-176 on d0 and d1, secondary abiotic IL-10^−/−^ mice were treated with resveratrol (RESVE; white boxes) or placebo (PLC; grey boxes) via the drinking water. On d6 post-infection, (**A**) macrophages and monocytes (F4/80^+^), (**B**) T lymphocytes (CD3^+^) and (**C**) B lymphocytes (B220^+^) were assessed microscopically from six high-power fields (HPF, 400× magnification) per animal in immunohistochemically stained colonic paraffin sections. Furthermore, (**D**) nitric oxide concentrations were measured in colonic ex vivo biopsies. Naive mice served as untreated and uninfected controls. Box plots represent the 75th and 25th percentiles of the median (black bar inside the boxes). Total range, significance levels (*p* values as determined by the one-sided ANOVA test followed by Tukey correction or by the Kruskal–Wallis test followed by Dunn’s correction) and numbers of mice (in parentheses) are given. Data were pooled from three independent experiments.

We further addressed whether resveratrol treatment was associated with less pronounced *C. jejuni* induced immune responses. We therefore quantified distinct innate as well as adaptive immune cell populations following immunohistochemical stainings of colonic paraffin sections with defined antibodies. At d6 following *C. jejuni* infection, markedly increased numbers of innate immune cell subsets such as F4/80^+^ macrophages and monocytes as well as of adaptive immune cell populations including CD3^+^ T lymphocytes and B220^+^ B cells could be observed in the colonic mucosa and lamina propria (*p* < 0.05–0.001; Figure 3A–C, Supplementary Figure S4B–D). In resveratrol-treated mice, however, colonic numbers of either immune cell subsets were lower at d6 p.i. as compared to placebo controls (*p* < 0.05–0.01; Figure 3A–C, Supplementary Figure S4B–D). Less pronounced increases in large intestinal mucosal immune cells were paralleled by less *C. jejuni* induced secretion of nitric oxide in colonic ex vivo biopsies obtained from resveratrol- as compared to placebo-treated mice at d6 p.i. (*p* < 0.05; Figure 3D). Hence, resveratrol treatment resulted in less pronounced *C. jejuni* induced pro-inflammatory immune responses in the large intestinal tract.

### 3.6. Colonic Epithelial Barrier Function of Resveratrol-Treated Mice Suffering from C. jejuni Induced Enterocolitis

**Figure 4:**
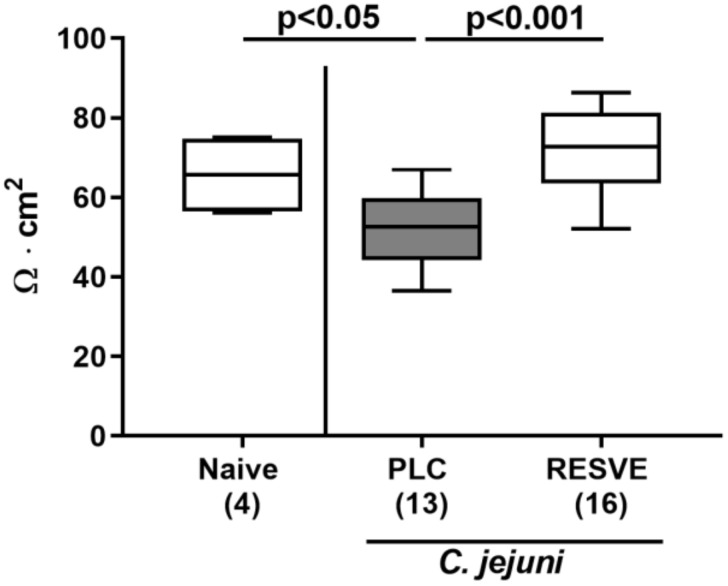
Colonic transmural electrical resistance following resveratrol treatment of *C. jejuni* infected mice. Starting two days after initial peroral infection with *C. jejuni* strain 81-176 on d0 and d1, secondary abiotic IL-10^−/−^ mice were treated with resveratrol (RESVE; white boxes) or placebo (PLC; grey boxes) via the drinking water. On d6 post-infection, the transmural electrical resistance (Rt) of the distal colon was measured in Ussing chambers (in Ω∙cm^2^). Naive mice served as untreated and uninfected controls. Box plots represent the 75th and 25th percentiles of the median (black bar inside the boxes). Total range, significance levels (*p* values as determined by the one-sided ANOVA test followed by Tukey correction) and numbers of mice (in parentheses) are given. Data were pooled from two independent experiments.

We further addressed whether resveratrol treatment could rescue epithelial barrier function following *C. jejuni* infection. Therefore, we performed electrophysiological resistance measurements of colonic ex vivo biopsies in the Ussing chamber. In fact, transmural resistances were lower in the large intestines derived from placebo, but not resveratrol-treated mice, at d6 p.i. as compared to naive controls (*p* < 0.05 versus naive, *p* < 0.001 versus resveratrol; Figure 4). Hence, resveratrol treatment could effectively rescue colonic epithelial barrier function following *C. jejuni* infection.

### 3.7. Extra-Intestinal Including Systemic Pro-Inflammatory Responses in Resveratrol-Treated Mice Suffering from C. jejuni Induced Enterocolitis

**Figure 5:**
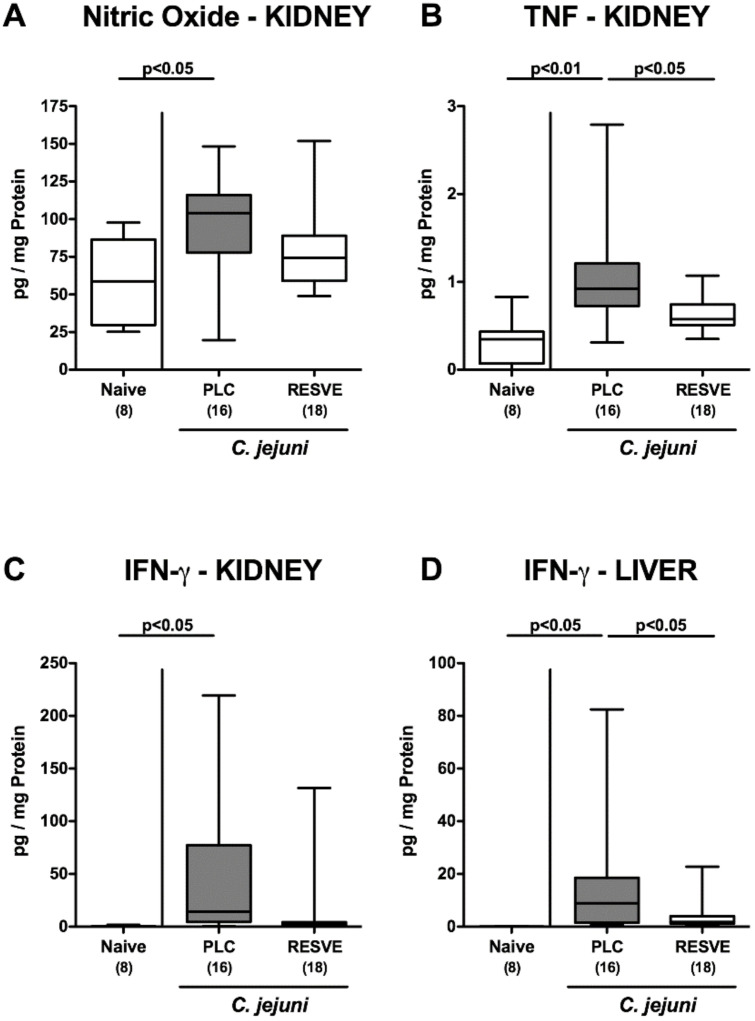
Inflammatory mediator secretion in extra-intestinal compartments following resveratrol treatment of infected mice suffering from acute enterocolitis. Starting two days after initial peroral infection with *C. jejuni* strain 81-176 on d0 and d1, secondary abiotic IL-10^−/−^ mice were treated with resveratrol (RESVE; white boxes) or placebo (PLC; grey boxes) via the drinking water. On d6 post-infection, extra-intestinal concentrations of (**A**) nitric oxide, (**B**) TNF-α and (**C**,**D**) IFN-γ were measured in ex vivo biopsies derived from kidneys (**A**–**C**) and the liver (**D**). Naive mice served as untreated and uninfected controls. Box plots represent the 75th and 25th percentiles of the median (black bar inside the boxes). Total range, significance levels (*p* values as determined by the one-sided ANOVA test followed by Tukey correction) and numbers of mice (in parentheses) are given. Data were pooled from three independent experiments.

Next, we addressed whether the disease-alleviating effects of resveratrol treatment were restricted to the intestinal tract or also effective in extra-intestinal compartments. Therefore, we measured pro-inflammatory mediators in ex vivo biopsies taken from kidneys and the liver. In fact, increased nitric oxide, TNF-α and IFN-γ concentrations could exclusively be assessed in the kidneys of *C. jejuni* infected mice from the placebo cohort (*p* < 0.05–0.001 versus naive; Figure 5A–C), which also held true for hepatic IFN-γ levels (*p* < 0.05; Figure 5D).

**Figure 6:**
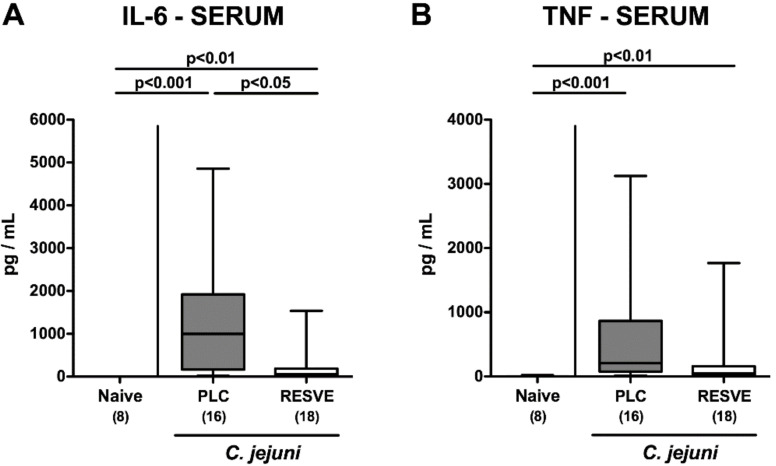
Systemic pro-inflammatory mediator secretion following resveratrol treatment of infected mice suffering from acute enterocolitis. Starting four days prior peroral infection with *C. jejuni* 81-176 strain on d0 and d1, secondary abiotic IL-10^−/−^ mice were treated with resveratrol (RESVE; white boxes) or placebo (PLC; grey boxes) via the drinking water. On d6 post-infection, serum concentrations of (**A**) IL-6 and (**B**) TNF-α were measured. Naive mice served as untreated and uninfected controls. Box plots represent the 75th and 25th percentiles of the median (black bar inside the boxes). Total range, significance levels (*p* values; as determined by the Kruskal–Wallis test followed by Dunn’s correction) and numbers of mice (in parentheses) are given. Data were pooled from three independent experiments.

Strikingly, resveratrol-mediated dampening of *C. jejuni* induced disease could also be observed systemically given that serum IL-6 concentrations were lower following resveratrol as compared to placebo treatment at d6 p.i. (*p* < 0.05; Figure 6), whereas a trend towards lower TNF-α concentrations could be observed in sera of the former as compared to the latter (not significant due to high standard deviations; Figure 6B). Hence, resveratrol treatment not only alleviated intestinal responses, but also extra-intestinal including systemic *C. jejuni* induced inflammatory responses.

## 4. Discussion

In our present preclinical intervention study we show, for the first time, that synthetic resveratrol application in the here-applied murine acute campylobacteriosis model (i) slightly lowered large intestinal luminal *C. jejuni* loads, (ii) resulted in a better clinical outcome that was accompanied by (iii) less distinct apoptosis of colonic epithelial cells and (iv) less pronounced pro-inflammatory immune responses in intestinal, extra-intestinal and, strikingly, systemic compartments and, furthermore, (v) in preserved colonic epithelial barrier function.

The observed disease-alleviating effects of resveratrol treatment were rather due to the amplified immune response than antimicrobial effects. Resveratrol has been shown to exert bacteriostatic properties against both Gram-positive and Gram-negative bacteria such as *Staphylococcus aureus* and *Escherichia coli*, respectively [26,27]. However, the underlying mechanisms are not yet fully understood. Changes in bacterial DNA content and disruption of bacterial membrane formation have been proposed, for instance [26,27,28]. Furthermore, resveratrol could effectively inhibit the adhesion of enteropathogens such as *Salmonella enterica* subspecies *enterica* serovar Typhimurium and enteropathogenic *E. coli* to colonic epithelial cell lines [29], whereas the polyphenolic compound could also antagonize the adhesive properties of *C. jejuni* to both biotic and abiotic surface structures [30]. Moreover, resveratrol prevented biofilm formation by *C. jejuni*, *E. coli* and *Aliarcobacter* species even when applied in concentrations below the MIC [31]. A slight reduction in the intestinal pathogen burden of one order of magnitude was observed in our present study, where the concentration of the applied resveratrol solution (300 mg/L) was slightly higher than the resveratrol MIC_90_ values (i.e., 228.25 mg/L) determined for 20 *C. jejuni* isolates, but below the resveratrol MIC of the *C. jejuni* strain 81-176 determined in vitro. Further, one needs to take into consideration that, following peroral application, resveratrol is rapidly metabolized in the body, and despite an absorption rate reaching 70%, the oral bioavailability needs to be considered as rather low [18,32]. Given that peroral resveratrol is also metabolized by the resident gut microbiota, it is unknown whether the observed effects are due to resveratrol alone and/or its metabolites [33].

The better clinical outcome of *C. jejuni* induced acute disease as indicated by less severe wasting and bloody diarrhea following resveratrol versus placebo application was accompanied by less pronounced colonic epithelial apoptosis and less colonic, extra-intestinal and systemic secretion of pro-inflammatory mediators. In support, a recent study applying a rabbit model of acute pharyngitis revealed that the anti-inflammatory effects of resveratrol were due to down-regulated caspase-3 expression and decreased IL-6 and TNF-α serum levels [34]. Resveratrol could effectively modulate both innate and adaptive immune responses upon *C. jejuni* infection, given that numbers in macrophages and monocytes as well as of T and B lymphocytes in the colonic mucosa and lamina propria were lower following resveratrol as compared to placebo application. These results are well in line with a previous in vitro study demonstrating that resveratrol impaired GM-CSF production [35] mounting in decreased differentiation, activation and survival of pro-inflammatory macrophages [36]. Furthermore, resveratrol application resulted in a dampened production of IFN-γ and TNF-α by activated macrophages and T lymphocytes in vitro [37,38]. In line, our group recently demonstrated that peroral resveratrol treatment of mice led to diminished T cell responses upon *Toxoplasma gondii* infection and subsequently prevented the animals from developing peracute ileitis [39]. In our present study, *C. jejuni* infected mice from the resveratrol cohort did not only exhibit decreased T cell, but also B cell numbers in their colonic mucosa and lamina propria. In support, resveratrol was shown to inhibit B cell proliferation in a murine model of systemic lupus erythematosus [40]. The diminished innate and adaptive immune cell responses upon resveratrol treatment of *C. jejuni* infected mice here were accompanied by reduced colonic secretion of nitric oxide, hence diminishing oxidative stress to the large intestinal epithelia and resulting in a preserved epithelial barrier function. Moreover, the reduction in epithelial apoptosis can provide an argument for a direct improvement in the transmural resistance in the colon of the infected and resveratrol-treated mice, since it was shown in vitro that apoptosis induction by *C. jejuni* is barrier-relevant and has the main impact on the epithelium in this context, besides tight junction changes [5]. The inhibition of apoptosis induction could restore barrier function in *C. jejuni* infection [5]. In support, resveratrol was shown to inhibit nitric oxide secretion from LPS-stimulated macrophages in vitro [41] and to reduce oxidative stress and inflammation by dampening the expression of the inducible nitric oxide synthase (iNOS) in vivo [42]. Moreover, resveratrol application to broilers could effectively ameliorate stress-induced impairment of intestinal barrier function [43].

Notably, in our intervention study resveratrol treatment did not only alleviate pro-inflammatory immune responses in the intestinal tract, but also in extra-intestinal and even systemic compartments of *C. jejuni* infected mice given attenuated pro-inflammatory cytokine secretion in the kidneys, the liver and the systemic blood stream. Our results are supported by multiple studies reporting potent anti-inflammatory effects of resveratrol in the kidneys, as shown in rat mesangial cell and human renal epithelial cells in vitro, for instance, and in several renal in vivo inflammation models as recently reviewed by Den Hartogh and colleagues [44]. Furthermore, anti-inflammatory and anti-oxidant effects of resveratrol were described in hepatic ischemic reperfusion injuries in diabetic rats [45] and in experimental as well as in clinical non-alcoholic fatty liver disease [46,47]. In addition, pronounced systemic disease-alleviating effects of resveratrol could be assessed in experimental systemic lupus erythematosus [40].

It is well known that the immunopathological sequelae during campylobacteriosis are caused by TLR-4-dependent host immune responses that are induced by LOS derived from the bacterial cell wall [11,48]. Since resveratrol has been shown to interact with TLR-4 [49] and to alleviate inflammation-induced cell damage in vascular endothelial cells by suppressing the TLR-4-dependent nuclear factor (NF)-κB signaling pathway [50], this suggests another health-beneficial mode of resveratrol action when applied in murine campylobacteriosis.

## 5. Conclusions

In conclusion, our preclinical intervention study provides strong evidence, for the first time, that synthetic resveratrol might be a promising candidate molecule to treat patients suffering from severe campylobacteriosis.

Long-term clinical trials revealed that resveratrol was well-tolerated by the individuals with doses of up to 5 g per day [51,52] and might therefore be considered as safe. Further studies either with resveratrol alone or in combination with other polyphenolic and/or non-polyphenolic compounds need to be performed in the future.

## Supplementary Materials

The following are available online at https://www.mdpi.com/2076-2607/8/12/1858/s1, Figure S1: Distribution of minimal inhibitory concentrations (MICs) of resveratrol among 20 *C. jejuni* isolates (in mmol/L and mg/L). The resveratrol MIC_90_ value indicates the resveratrol concentration resulting in inhibition growth of 90% of the tested *C. jejuni* isolates; Figure S2. Fecal *C. jejuni* shedding over time following resveratrol treatment of infected mice suffering from acute enterocolitis; Figure S3. Clinical conditions over time following resveratrol treatment of infected mice suffering from acute enterocolitis; Figure S4. Representative photomicrographs illustrating colonic apoptotic and immune cell responses following resveratrol treatment of infected mice suffering from acute enterocolitis.

## Abbreviations

CBA	cytometric bead array
CFU	colony forming units
CLSI	Clinical and Laboratory Standards Institute
HPF	high-power fields
IFN	interferon
IL	interleukin
iNOS	inducible nitric oxide synthase
LOS	lipooligosaccharide
LPS	lipopolysaccharide
MIC	minimal inhibitory concentration
NF	nuclear factor
PBS	phosphate-buffered saline
p.i.	post-infection
R^t^	transmural electrical resistance
TLR	Toll-like receptor
TNF-α	tumor necrosis factor-α
Th-1 cells	T helper-1 cells
