# Supplementary material for: Resveratrol Alleviates Acute Campylobacter jejuni Induced Enterocolitis in a Preclinical Murine Intervention Study
Source: Microorganisms. 2020 Nov 25;8(12):1858. doi: 10.3390/microorganisms8121858 (PMC7760181; doi:10.3390/microorganisms8121858)
Supplement: Supplementary file 1 [file microorganisms-08-01858-s001.pdf]

Supplementary Materials

RESVERATROL	MIC: mmol/L (mg/L)	0.008 (1.78)	0.0156 (3.56)	0.0313 (7.13)	0.0625 (14.26)	0.125 (28.53)	0.25 (57.06)	0.5 (114.13)	1.0 (228.25)	2.0 (456.5)	4.0 (913.0)	8.0 (1826.0)	MIC90: mmol/L (mg/L)	1.0 (228.0)
C. jejuni	Number of isolates	0	0	0	0	0	2	1	15	2	0	0		

Figure S1: Distribution of minimal inhibitory concentrations (MIC) of resveratrol among 20 *C. jejuni* isolates (in mmol/L and mg/L). The resveratrol MIC_{90} value indicates the resveratrol concentration resulting in inhibition growth of 90% of the tested *C. jejuni* isolates;

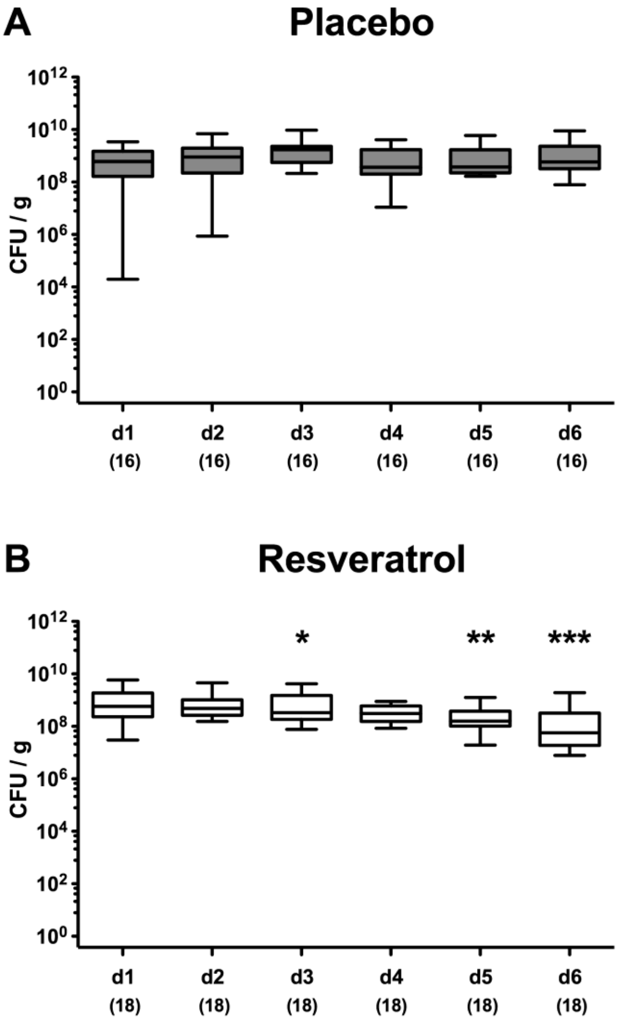

Figure S2. Fecal *C. jejuni* shedding over time following resveratrol treatment of infected mice suffering from acute enterocolitis.

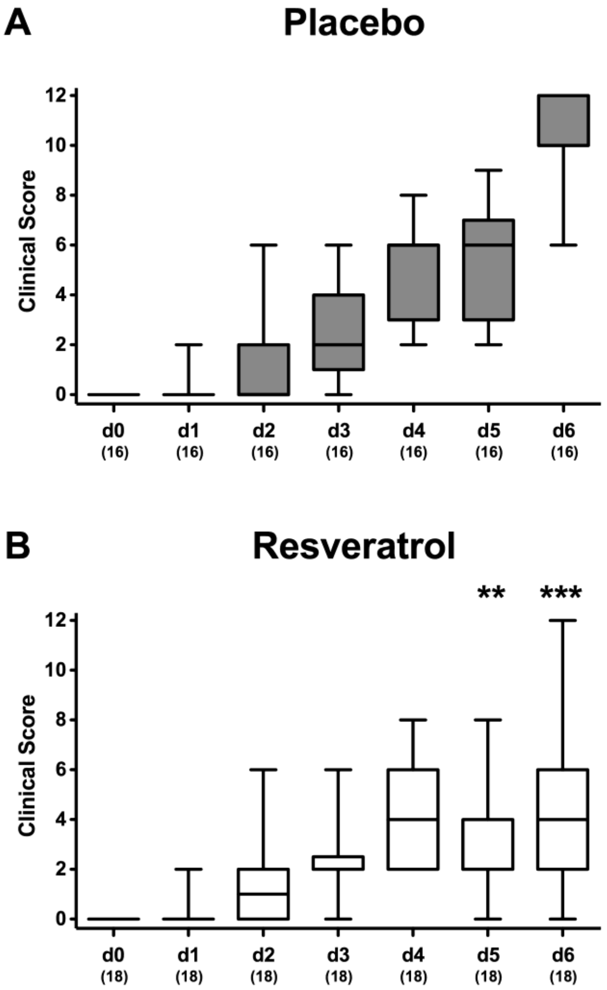

Figure S3. Clinical conditions over time following resveratrol treatment of infected mice suffering from acute enterocolitis.

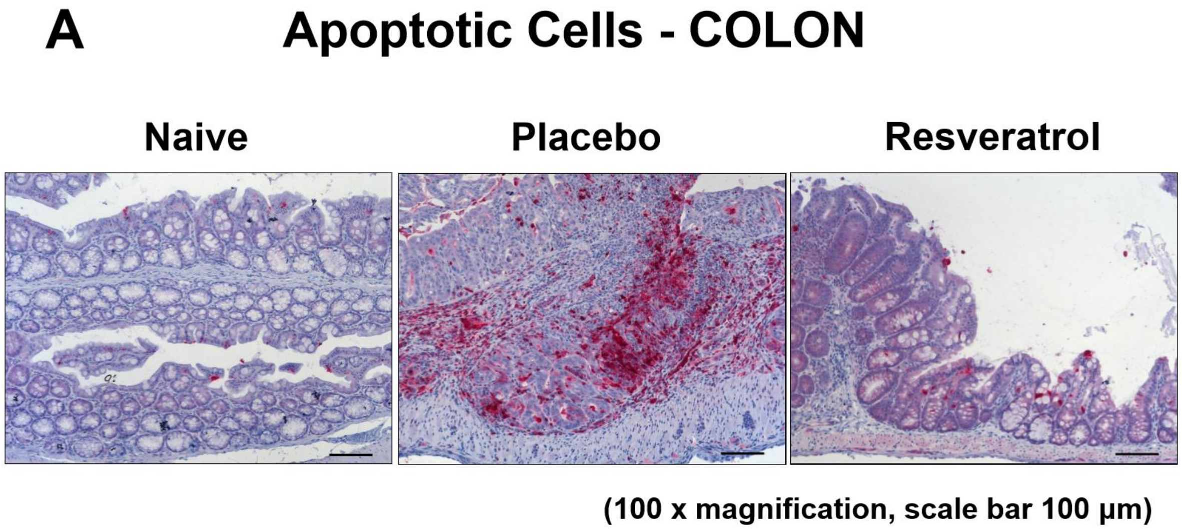

Figure S4. Representative photomicrographs illustrating colonic apoptotic and immune cell responses following resveratrol treatment of infected mice suffering from acute enterocolitis.
